# Disseminated mycobacterium genavense infection with central nervous system involvement in an HIV patient: a case report and literature review

**DOI:** 10.1186/s12879-024-09316-x

**Published:** 2024-04-24

**Authors:** Ali Hassanzadeh, Malihe Hasannezhad, Ladan Abbasian, Sara Ghaderkhani, Fereshteh Ameli, Mehdi Allahdadi

**Affiliations:** 1 Department of Infectious Diseases and Tropical Medicine, Imam Khomeini Hospital Complex, Tehran University of Medical Sciences, End of Keshavarz Boulevard, 1419733141 Tehran, Iran; 2 Iranian Research Center for HIV/AIDS, Imam Khomeini Hospital Complex, Tehran University of Medical Sciences, Tehran, Iran; 3 Department of Pathology, School of Medicine, Tehran University of Medical Sciences, Tehran, Iran

**Keywords:** Mycobacterium genavense, HIV, Central nervous system, Case report

## Abstract

**Background:**

Immunodeficient patients, particularly HIV patients, are at risk of opportunistic infections. Nontuberculous mycobacteria can cause severe complications in immunodeficient patients.

**Case Presentation:**

We describe a 57-year-old HIV patient, primarily presented with coughs and constitutional symptoms, with a unique *Mycobacterium genavense* abdominal, pulmonary, and central nervous system infection, accompanied by intracranial masses.

**Conclusion:**

The diagnosis of NTM, including *M. genavense*, must always be considered by clinicians in immunodeficient patients, especially those with HIV, who have a compromised immune system.

**Supplementary Information:**

The online version contains supplementary material available at 10.1186/s12879-024-09316-x.

## Background

Nontuberculous mycobacterial (NTM) infections are a major concern for HIV-infected patients. Their main habitats in the environment are water sources and dust [1] and they can infect multiple organs in the host [2]. *Mycobacterium genavense* (*M. genavense*) is reported to be responsible for more than 10% of disseminated NTM infections [3, 4]. Abdominal organs, including lymph nodes, liver, spleen and gastrointestinal tracts are the main targets for *M. genavense* infection [5]. Despite the improvements in survival by antiretroviral therapies (ART) in the recent years, the prognosis of this infection remains poor [5] due to the long treatment periods and high prevalence of side effects, plus non-specific diagnostic and treatment tools [6–8].

The disseminated presentation of the infection has been repeatedly reported across the world; however, involvement of the central nervous system (CNS) is rarely observed. In this paper, we will discuss a case admitted to our center and review the published literature to investigate the diagnostic means of this unique form of infection.

## Case presentation

**Fig. 1:**
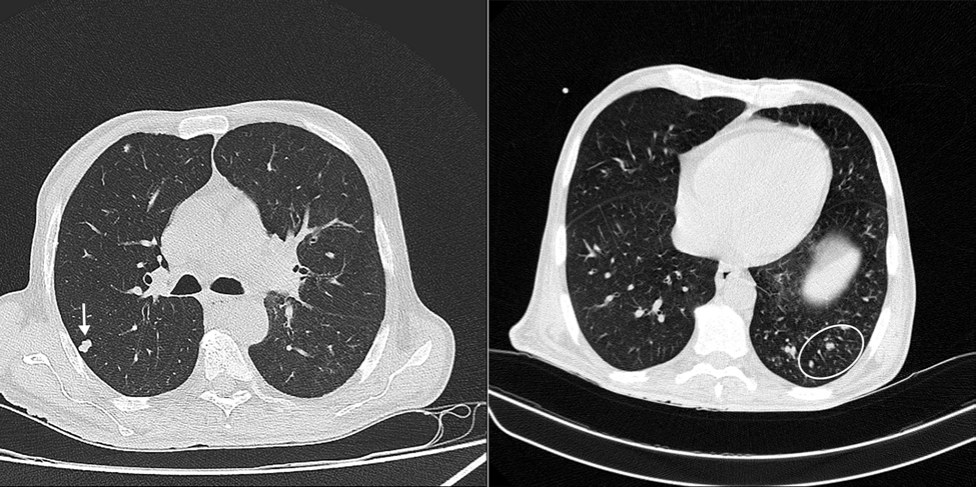
Pulmonary nodule (white arrow) and tree-in-bud pattern (circled area) in the chest CT scan

Our patient is a 57-year-old male, with a four-month history of HIV infection (CD4 count = 10/µL, HIV viral load > 3,000,000 copies/ml) and cytomegalovirus (CMV) retinitis, who complained of worsening fatigue and nausea for two months. He also complained of progressive unintentional weight loss (over 30 kg in this period) and a productive cough. No vision or sensory symptoms was reported. Additionally, there was no evidence of fever, loss of consciousness, or cognitive-behavioral demonstration; however, he had given a history of disrupted gait. Although, the neurological (i.e., finger-to-nose, heel-to-shin, and limb forces) and meningitis tests (i.e., neck stiffness, Brudzinski’s and Kernig’s signs) were normal. This could be related to the severe generalized weakness, as no neurological deficit was identified. He was on a drug regimen consisting of Truvada® (emtricitabine 200 mg– tenofovir disoproxil 300 mg) q24h, dolutegravir 50 mg q24h, and valganciclovir 450 mg q12h and was compliant with treatment. Laboratory results showed a pancytopenia (WBC = 1,500/µL, Hb = 6 g/dL, and platelets = 125,000/µL) and elevated CRP level of 92 mg/L. Lung CT-scan showed a 9 mm×6 mm nodule in the right middle lobe and a tree-in-bud pattern at the lower levels of the left lung (Fig. 1). Abdominal ultrasound investigation revealed an enlarged spleen (15.5 cm), multiple enlarged paraaortic lymph nodes, and a 7 mm lymph node in the *porta hepatis*.

**Fig. 2:**
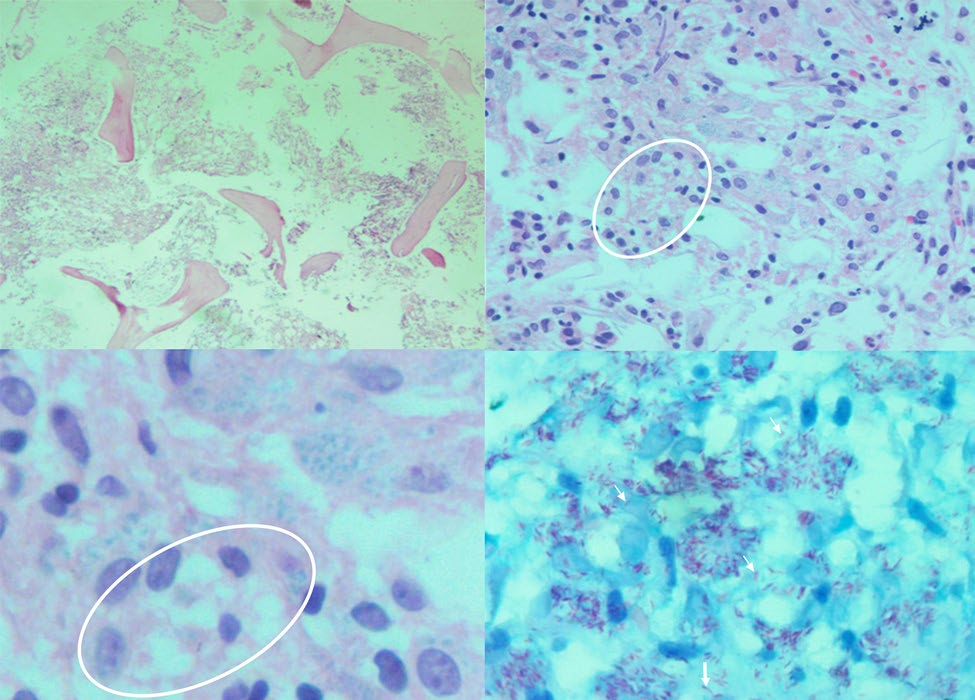
Pathology sections of bone marrow biopsy specimen; Low magnification of bone marrow biopsy shows replacement of hematopoietic elements by numerous foamy macrophages (circled area) arranged in sheet (H&E section, x40 & x100). Higher magnification reveals histiocytes (circled area) containing abundant organisms (H&E section x400). Frequent positive acid-fast bacilli (white arrows) were present in foamy macrophages on Ziehl-Nielsen stain (x400)

**Fig. 3:**
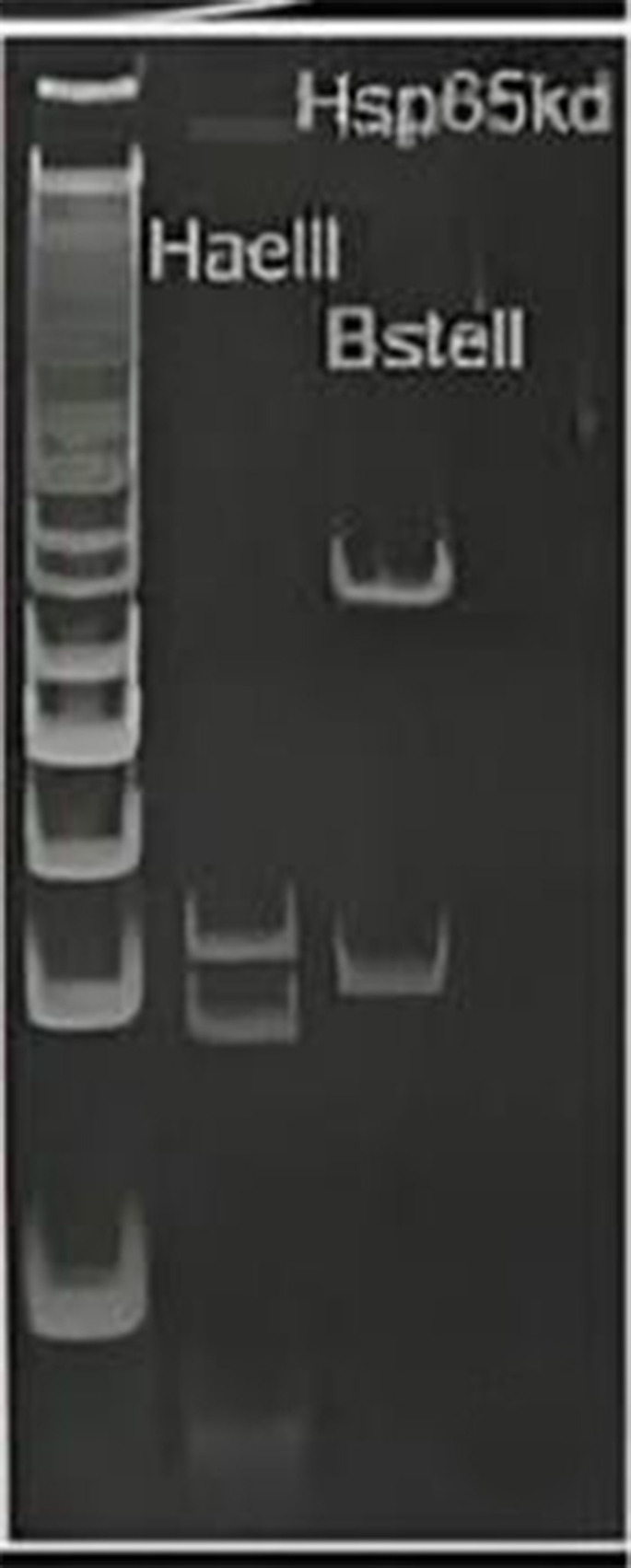
*hsp65* gene sequencing

A cerebrospinal fluid (CSF) analysis indicated a decreased glucose level (24 mg/dL) as well as a normal protein level of 28 mg/dL and a WBC count of 0–1/L. Acid fast bacilli (AFB) staining in both sputum and CSF was positive. Viral, namely CMV and varicella zoster virus, and fungal diagnostic tests on the CSF specimen for probable agents were negative. No pathological finding in brain MRI was observed at this stage. Considering *Mycobacterium tuberculosis* (MTB) infection, we started an anti-MTB empirical treatment, consisting of isoniazid, pyrazinamide, ethambutol, and rifampin (liver function tests were normal). Due to the pharmacokinetic interactions of rifampin and dolutegravir [9], we increased the dose of dolutegravir to 50 mg q12 h. No MTB growth was observed in the culture and GeneXpert® molecular tests of sputum and CSF were negative for MTB, raising the probability of NTM infection. Due to low blood cell counts, lymphadenopathy, and splenomegaly, we performed a simultaneous bone marrow biopsy and observed foamy histiocytes and NTM presence (AFB staining positive and GeneXpert® negative) (Fig. 2). Clarithromycin was added to the previous regimen, for NTM infection coverage. At this stage, the results of previously-requested NTM polymerase chain reaction (PCR) analysis from the CSF specimen detected *M. genavense* presence (complete match to *hsp65* gene) (Fig. 3). Considering the NTM infection in the respiratory system and bone marrow, and *M. genavense* meningitis, we made a disseminated *M. genavense* infection diagnosis. The patient was discharged with NTM combinational drug treatment (five-drug) accompanied by ART and valganciclovir as his medical condition was stable.

**Fig. 4:**
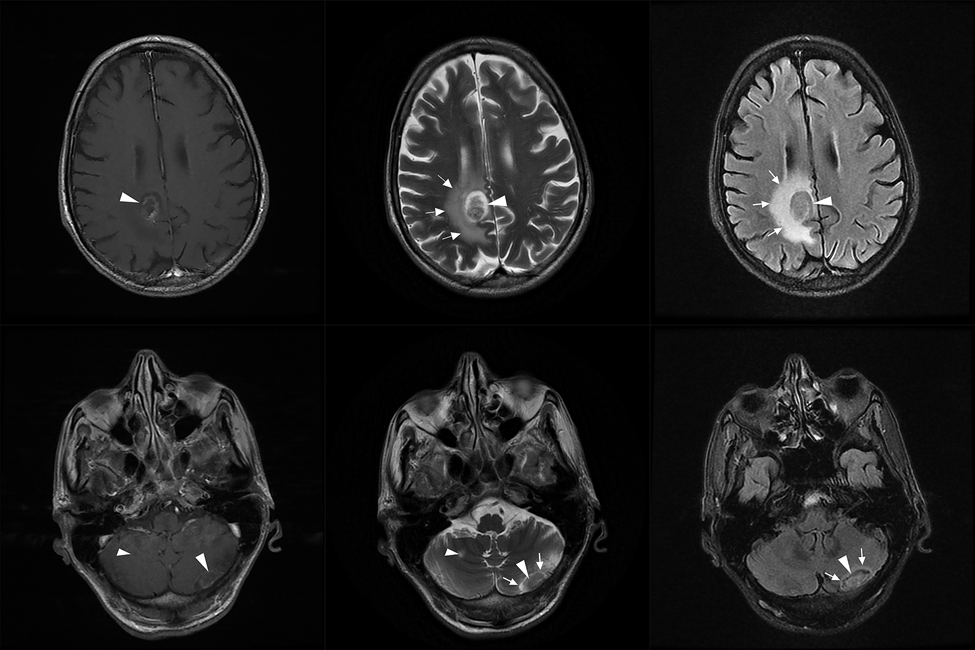
Brain MRI reveals a right sided mass (white arrowheads) in the corpus callosum with perilesional edema (white arrows) and mass effects on the ventricle, and two lesions (white arrowheads) in the hemispheres of cerebellum accompanied by edema (white arrows). [Left to right: T1 with gadolinium contrast, T2, and FLAIR views]

**Fig. 5:**
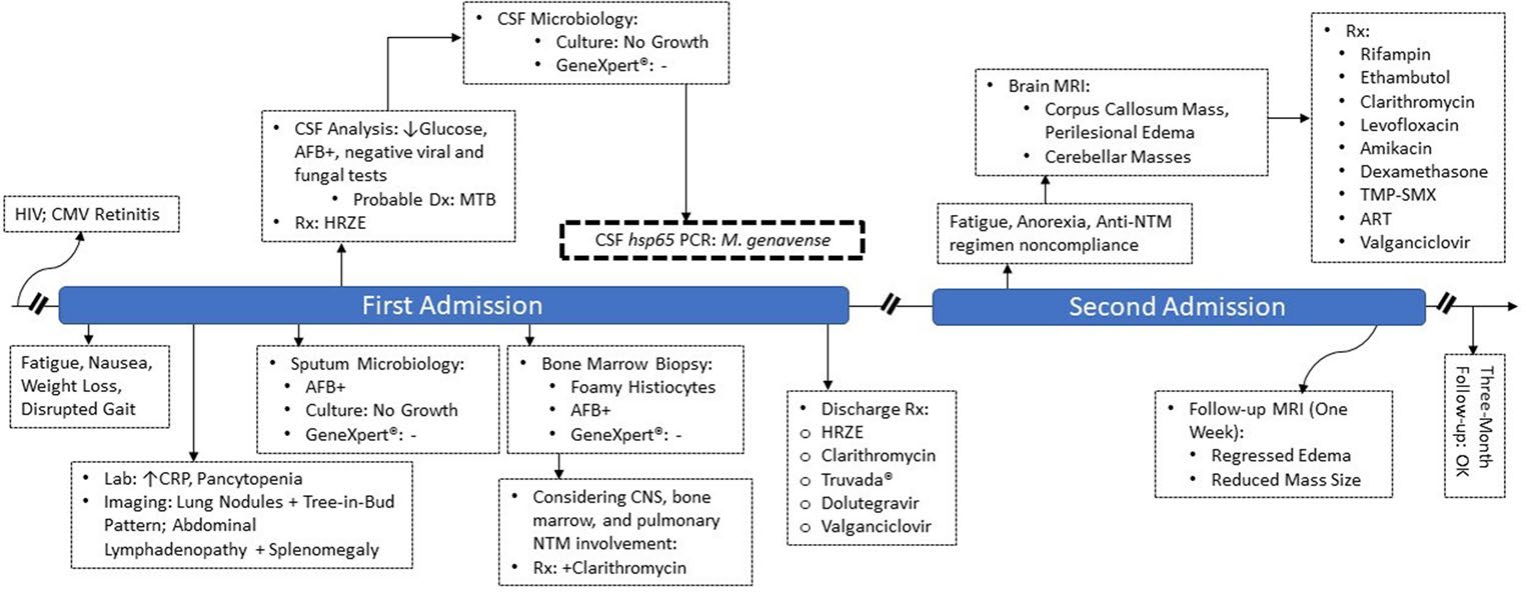
Clinical course of the patient (HRZE: anti-TB therapy combination including isoniazid, rifampin, pyrazinamide, and ethambutol; TMP-SMX: trimethoprim-sulfamethoxazole

Four months later he presented with generalized fatigue and anorexia, while claiming he had not consumed the NTM prescription appropriately; however, he was compliant with ART and valganciclovir. There were no abnormal findings in the examinations. CD4 count was 13/µL and lung CT scan prevailed that the tree-in-bud pattern vanished but the nodule was present with no significant size change. According to his previous history, we requested a brain MRI, which showed a right hemispherical mass in the corpus callosum with mass effects on the ventricle, accompanied by edema and two lesions in both hemispheres of the cerebellum (Fig. 4). The patient did not consent for a cerebral biopsy to investigate potential diagnoses, such as toxoplasmosis and malignancies. However, considering the previously confirmed presence of *M. genavense* in CNS and poor Anti-NTM regimen compliance, the intracranial masses were most likely formed in the background of disseminated *M. genavense* infection. Anti-NTM drug combinations (ethambutol 1,200 mg q24h, rifampin 600 mg q24h, clarithromycin 500 mg q12h, levofloxacin 750 mg q24h, and amikacin 1,000 mg q24h) were initiated in conjunction with ART, valganciclovir, and prophylactic trimethoprim-sulfamethoxazole. We started dexamethasone eight mg q12h for perilesional edema. The next MRI within one week showed that the edema had regressed and reduced cerebral mass size, favoring the diagnosis. After the treatment, his clinical progression was desirable and he was discharged with the same prescriptions as of admission (except for amikacin, which was discontinued due to a rise in serum creatinine up to 1.8 mg/dL, and then improved before discharge to baseline by discontinuing the drug and hydrating). Dexamethasone was replaced by prednisolone, which was tapered gradually over the following weeks. Three months later, his symptoms were relieved, his drug compliance was complete, and he was clinically stable (Fig. 5).

## Discussion and conclusions

*M. genavense* is one of the most common causes of mycobacterial infections in avians, especially parrots [10]. Colonization of *M. genavense* in the human body is common and almost always does not result in disease; however, a case of disseminated infection has recently been published in a previously healthy pet keeper, hypothesizing the zoonotic transmission probability [11]. As a widespread family, NTM could be isolated from various specimens due to colonization or specimen contamination [12, 13]. *M. genavense* has been detected more frequently in HIV patients [5, 12]. Despite this fact, multiple reports have discussed *M. genavense* infection in non-HIV immunodeficiencies, namely sarcoidosis, solid organ recipients, and primary immunodeficiencies [14–17]. Diagnostic and treatment challenges are the most significant challenges in disease management.

As well as constitutional symptoms, *M. genavense* commonly manifests symptoms involving the gastrointestinal and abdominal organs, including abdominal pain, hepatosplenomegaly, and lymphadenopathy [3, 5]. Thomsen et al. have hypothesized that more frequent abdominal manifestations might be a result of the presence of the microorganism in the GI tract of the infected [18]. Only a few cases with *M. genavense* CNS involvement have been reported worldwide. After a systematic literature review in PubMed, Embase, and Web of Science online databases with ‘Genavense’ AND ‘HIV’ keywords, we have identified seven cases with background immunodeficiency plus CNS *M. genavense* infection. Five cases were HIV infected [19–23] and the remaining two were primary immunodeficiency cases (including a case of hypogammaglobulinemia [24] and an Adenosine Deaminase deficiency patient with a history of gastrointestinal *M. genavense* infection [25]). Table 1 provides the main clinical and laboratory characteristics of HIV cases with *M. genavense* CNS involvement.

**Table 1:** Clinical and laboratory characteristics of HIV patients with *M. genavense* CNS involvement

First author, Y (ref)	Country	Age, Sex	HIV diagnosis	Primary manifestations	Previous significant History	Physical examination		Imaging		Laboratory/Pathology		
						Neurological	Other	Brain MRI/CT	Other	CD4 count /µL	CSF	Biopsy ± Microbiology
Berman, 1994 [19]	USA	50, M	7 months	Seizure, right-sided paresthesia/decreased force, memory issues	N/A	Unilateral sensorimotor deficits	Kaposi’s sarcoma in lower limbs, molluscum contagiosum lesions	Left parietal mass	N/A	84	N/A	**Mass**: [*Bx*: lymphohistiocytic infiltrations, *AFB*: (+), *culture*: *M. genavense*] **BM**: [*Bx*: no granulomatous infection]
Kyrill, 2013 [20]	Belgium	58, F	15 years	Fever, weight loss, diarrhea, hypotension	No ART compliance	Decreased DTRs, gait disturbance	Hepatosplenomegaly	N/A	Infarcted spleen, bone marrow and lymph nodes involvement	14	*culture and 16s-rRNA: M. genavense*	**BM**: [*Bx*: non-diagnostic, *AFB*: (+), *culture*: *M. genavense*] *Blood culture*: *M. genavense*
Toussi, 2017 [21]	USA	39, F	N/A	Work-up for intracranial mass, constitutional	N/A	Normal	N/A	Right parietal mass	Lung nodule (unknown origin)	N/A	N/A	**Mass**: [*Bx*: epithelioid spindle-shaped histiocytes with reactive responses, *AFB*: (+), *rRNA-16s*: *M. genavense*]
Vazquez, 2022 [22]	Argentina	49, M	N/A	Left shoulder pain	Cryptococcosis, CMV, HZV, atypical mycobacterium (doudenal and bone marrow), nodular lesions in brain (2 years) responsive to anti-mycobacterial treatment	Normal	N/A	right temporo-parietal mass	Lung nodule, adjacent pleural thickening and rib osteolysis	100	N/A	**Mass**: [*AFB*: (+), *Bx*: N/A, *culture*: atypical mycobacterium, *rRNA-16s*: *M. genavense*]
Kuczynski, 2023 [23]	Canada	54, M	9 years	Focal seizure	No ART compliance (for 5 years)	Normal	N/A	left parietal mass	Ruled out malignancy	166	*AFB*: (-), mildly increased protein	**Mass**: [*Bx*: Langerhans giant cells and granulomatous inflammation, *AFB*: (+), *MTB & MAC PCR*: (-), *rRNA-16 S & hsp65*: *M. genavense*]
*Current Study*	Iran	57, M	4 months	Fatigue and nausea, weight loss, cough	CMV retinitis	Normal	Splenomegaly	First admission: no finding, F/U: Right hemispherical mass in the corpus callosum, edema, and two lesions in the cerebellum	Lung nodule, tree-in-bud pattern, paraaortic and porta hepatis lymphadenopathy	10, 13	*AFB*: (+), decreased glucose, *TB culture and GeneXpert®*: (-), *hsp65*: *M. genavense*	**BM**: [*Bx*: foamy histiocytes and atypical mycobacterium, *AFB*: (+)]

*Abbreviation* AFB: acid-fast bacilli, ART: antiretroviral therapy, BM: bone marrow, Bx: biopsy, CMV: cytomegalovirus, DTR: deep tendon reflex, F/U: follow-up, HZV: herpes zoster virus, MAC: mycobacterium avium complex, MTB: mycobacterium tuberculosis, N/A: not reported/not obtained

Our case is the sixth reported HIV case with *M. genavense* CNS involvement. Previously, the microorganism was isolated from the CSF of two cases [20, 23], and one developed a cerebral mass without other organ involvement [23]. Other three cases suffered from intracranial masses, but their CSF were not examined [19, 21, 22]. One of these patients had simultaneous lung nodule and pleural involvement [22], while the authors of the remaining studies did not address further involvement [19, 21]. The present case is a composite abdominal, pulmonary, and CNS infection caused by *M. genavense*, accompanied by intracranial lesions, which is unique. Disseminated *M. genavense* diagnosis was highly plausible in this case due to the synchronicity of M. genavense isolation from CNS, and pulmonary and bone marrow NTM involvement. Also, due to the history of CNS involvement and poor drug compliance, the intracranial masses are likely to be related to the *M. genavense* background; However further measures would be mandatory in the case of clinical course reverse or probable drug resistance. Interestingly, no neurological abnormality was present in physical examination in the second admission, similar to three of the previous cases [21–23]. This may originate from the chronic and insidious and chronic clinical progression of the NTM [26, 27]. Imaging and pathology beside molecular tests play a key role in *M. genavense* infection confirmation. However, the diagnosis should be highly concerned when NTM infection is present (when culture and TB molecular tests are inconclusive) in an immune-compromised patient. Microbiology tests must rely on molecular assessments [28], as the time to identification in fortified growth media could be as long as 91 days or more [14, 20, 29].

Recent introduction of ART has improved overall survival significantly; however, *M. genavense* could be lethal [5]. Treatment of the disease remains controversial. A recent individual patient meta-analysis concluded that macrolides might be related to lower fatalities; and other agents, such as amikacin, have no significant association with survival [5]. It has been shown that azithromycin, ethambutol, and rifampicin combination may be effective for *M. genavense* lung disease [28].

CNS infection treatment knowledge is restricted to previous experiences. Kuczynski et al. and Berman et al. initiated corticosteroid and an anti-NTM regimen without mass resection and the patients were stable after nine [23] and twelve months [19]. The Belgian case died roughly two weeks after treatment (clarithromycin, ethambutol, rifabutin, moxifloxacin, and amikacin) due to unsteady hemodynamics and consciousness [20]. Toussi et al. performed lesion resection due to high pre- and intraoperative malignancy suspicion and no follow-up was provided [21]. Another case presented with pulmonary involvement of *M. genavense* with an incidental, asymptomatic intracranial mass. The patient underwent surgical lesion excision; nevertheless, his condition worsened and he died due to respiratory failure [22]. Our patient was primarily diagnosed with *M. genavense* meningitis with no pathologic findings on imaging. However, he developed multiple intracranial masses after months. Medication noncompliance might be a reason for this progression, and one reason is the high quantity of medications [30, 31]. The NTM infection has a poor prognosis, with a mortality rate estimated at 32–39.3% among HIV patients [5, 12], which illustrates the pathogen’s invasiveness and ineffective treatment methods. A recent systematic review of NTM CNS infections reported a 37.5% case fatality rate; although this review did not include any *M. genavense* cases [32].

Clinicians must always consider the diagnosis of NTM, including *M. genavense*, in immunodeficient patients, especially those with HIV. The outcomes remain unfavorable despite ART and antibiotic developments. Due to the low prevalence of the disease, no consensus management of CNS involvement is available. As we have reviewed, invasive CNS treatment must be decided according to medical status due to possible lack of effectiveness. Macrolides, ethambutol, and rifamycins might improve disseminated infection outcomes and should be considered first-line treatment. Further multicenter prospective studies might identify poor outcomes predictors.

### Electronic supplementary material

Below is the link to the electronic supplementary material.

Supplementary Material 1

## Data Availability

Not applicable.

## Abbreviations

NTM: Nontuberculous Mycobacterium
ART: Antiretroviral Therapy
CNS: Central Nervous System
CMV: Cytomegalovirus
AFB: Acid Fast Bacilli
MTB: *Mycobacterium tuberculosis*
CSF: Cerebrospinal Fluid
PCR: Polymerase Chain Reaction
