# Supplementary material for: Disseminated mycobacterium genavense infection with central nervous system involvement in an HIV patient: a case report and literature review
Source: BMC Infect Dis. 2024 Apr 24;24:437. doi: 10.1186/s12879-024-09316-x (PMC11041032; doi:10.1186/s12879-024-09316-x)
Supplement: Supplementary file 1 — Supplementary Material 1 [file 12879_2024_9316_MOESM1_ESM.pdf]

CARE Checklist of information to include when writing a case-report

| Topic | Item | | Reported on Line: |
|---|---|---|---|
| Title | 1 | The diagnosis or intervention of primary focus followed by the words “case report” | 2-3 |
| Key Words | 2 | 2 to 5 key words that identify diagnoses or interventions in this case report, including "case report" | 42 |
| Abstract (no references) | 3a | Introduction: What is unique about this case and what does it add to the scientific literature? | 35-37 |
| | 3b | Main symptoms and/or important clinical findings | 37-38 |
| | 3c | The main diagnoses, therapeutic interventions, and outcomes | 37-38 |
| | 3d | Conclusion—What is the main “take-away” lesson(s) from this case? | 39-40 |
| Introduction | 4 | One or two paragraphs summarizing why this case is unique (may include references) | 50-51 |
| Patient Information | 5a | De-identified patient specific information | 55 |
| | 5b | Primary concerns and symptoms of the patient | 57-60 |
| | 5c | Medical, family, and psycho-social history including relevant genetic information | 55-57 |
| | 5d | Relevant past interventions with outcomes | Not Applicable |
| Clinical Findings | 6 | Describe significant physical examination (PE) and important clinical findings | 60-61 |
| Timeline | 7 | Historical and current information from this episode of care organized as a timeline | 55 & 77 & 82 |
| Diagnostic Assessment | 8a | Diagnostic testing (such as PE, laboratory testing, imaging, surveys) | 63-69 & 73-79 |
| | 8b | Diagnostic challenges (such as access to testing, financial, or cultural) | Not Applicable |
| | 8c | Diagnosis (including other diagnoses considered) | 79 (& 70 & 74) |
| | 8d | Prognosis (such as staging in oncology) where applicable | Not Applicable |
| Therapeutic Intervention | 9a | Types of therapeutic intervention (such as pharmacologic, surgical, preventive, self-care) | 70-73 |
| | 9b | Administration of therapeutic intervention (such as dosage, strength, duration) | 70-73 & 75 & 88-92 &93-95 |
| | 9c | Changes in therapeutic intervention (with rationale) | 72 & 93-95 |
| Follow-up and Outcomes | 10a | Clinician and patient-assessed outcomes (if available) | 79-81 & 82-83 & 92-96 |
| | 10b | Important follow-up diagnostic and other test results | 94-96 |

| | | | |
|---|---|---|---|
| | 10c | Intervention adherence and tolerability (How was this assessed?) | 82-83 & 95-96 |
| | 10d | Adverse and unanticipated events | 86-88 & 93-95 |
| Discussion | 11a | A scientific discussion of the strengths AND limitations associated with this case report | 116-122 |
| | 11b | Discussion of the relevant medical literature with references | 111-115 & 116-120 & 134-141 |
| | 11c | The scientific rationale for any conclusions (including assessment of possible causes) | 127-128 & 130-133 |
| | 11d | The primary "take-away" lessons of this case report (without references) in a one paragraph conclusion | 149-155 |
| Patient Perspective | 12 | The patient should share their perspective in one to two paragraphs on the treatment(s) they received | Not Applicable |
| Informed Consent | 13 | Did the patient give informed consent? Please provide if requested: | Yes; IR.TUMS.IKHC.REC.1402.276 |
